# Pan centromeric FISH enhances precision in radiation biodosimetry

**DOI:** 10.1038/s41598-025-34407-3

**Published:** 2026-02-10

**Authors:** Rajesh Kumar Chaurasia, Aarti Notnani, Devina Fenilon Vaz, Kapil B. Shirsath, Sheeri Fatima, Nagesh N. Bhat, Arshad Khan, Dhruv Kumar, Balvinder K. Sapra

**Affiliations:** 1 Radiological Physics and Advisory Division, Bhabha Atomic Research Centre (BARC), Mumbai, India; 2 School of Health Sciences and Technology, University of Petroleum and Energy Studies, Dehradun, India; 3 Homi Bhabha National Institute (HBNI), Mumbai, India

**Keywords:** Biodosimetry, Pan-centromeric FISH, Cytogenetic biodosimetry, Dose-response curves, Radiological emergencies, Chromosomal aberrations, Biophysics, Biomarkers, Health care, Health occupations

## Abstract

Accurate biodosimetry is critical for assessing radiation exposure in radiological emergencies, occupational monitoring, and clinical management, where precise dose estimation informs life-saving decisions and regulatory compliance. Current gold-standard cytogenetic methods face limitations in sensitivity and reproducibility, especially at low doses (< 0.5 Gy) (practical limitations at low doses, including very low dicentric yields, higher statistical noise, and greater scoring uncertainty as aberration frequencies near background levels). This study presents a systematic comparison of pan-centromeric fluorescence in situ hybridization (pan-cent-FISH) and Giemsa staining for detecting dicentric (DC) and ring (R) chromosomes following ^60^Co-γ irradiation (0–3 Gy). Analysis of more than 15,000 metaphases per technique revealed enhanced sensitivity of pan-cent-FISH technique, demonstrating a 1.72-fold higher linear coefficient and enhanced (1.13-fold) quadratic coefficient (β), indicating improved sensitivity across both low and high dose ranges. Blind validation with eight samples showed pan-cent-FISH achieved ~ 2-fold greater accuracy, with mean absolute differences of 0.0538 Gy (vs. 0.1105 Gy for Giemsa) and average relative errors of 7.13% (vs. 15.35% for Giemsa). At low doses (0.1 Gy), pan-cent-FISH maintained 9.0% error, while Giemsa exceeded acceptable limits (21.0% error). The standardized fluorescence detection used for the technique eliminated morphological ambiguities, reducing false negatives by ~ 40% and improving first-pass accuracy.

## Introduction

Accurate assessment of radiation exposure remains a critical challenge in both regulatory occupational monitoring and emergency response scenarios^1^. The biological consequences of ionizing radiation are intrinsically linked to the induction of chromosomal aberrations, with dicentric chromosomes (DCs) and ring chromosomes (Rs) serving as particularly reliable biomarkers due to their radiation-specific formation and dose-dependent frequency^2^. For decades, conventional uniform Giemsa staining has been the workhorse of cytogenetic biodosimetry, providing a cost-effective means of visualizing these aberrations^3^. However, the reliance of this technique on subjective morphological interpretation introduces significant limitations, particularly when analyzing complex aberrations in metaphase spread or when identifying centromere-related features of dicentric and ring chromosomes that often appear ambiguous. These challenges are further compounded by variable chromosome condensation, overlapping structures, and poorly resolved centromeres, factors known to increase false negatives and inter-observer variability in conventional Giemsa scoring^4,5^. Missing even a few such ambiguous dicentrics can significantly affect dose estimation at lower doses, where aberration yields are inherently low and the loss of any true DC has a significant impact on accuracy^1,2^. In contrast, pan-centromeric FISH overcomes many of these limitations by fluorescently labelling all centromeres, enabling unambiguous visualization of dual centromeres-including those that are closely spaced in DCs and permitting reliable detection even in poorly condensed chromosomes where uniform Giemsa staining cannot resolve centromeres precisely^4,5^. It also improves recognition of rings lacking clear morphology (where centromere is not clear) and supports accurate scoring in suboptimal or partially overlapping metaphases. This fluorescence-based clarity reduces observer dependency and false-negative rates, thereby enhancing sensitivity and dose-estimation precision^6^. These challenges become particularly acute in scenarios demanding high analytical precision, whether for monitoring radiation workers near regulatory dose limits, or managing large-scale radiological incidents where accurate triage is paramount^1,2^.

The emergence of fluorescence in situ hybridization (FISH) techniques has enabled targeted visualization of specific chromosomal regions^7^. Pan-centromeric FISH (pan-cent-FISH), which fluorescently labels all centromeres, offers a transformative solution to the limitations of conventional staining by providing unambiguous identification of radiation-induced aberrations^4^. This approach proves especially valuable for detecting DC and R (“DC + R”) that might be missed by uniform Giemsa staining - those with closely spaced centromeres, derived from acrocentric chromosomes, or present in crowded metaphase spreads^5^. The enhanced detection capability of pan-cent-FISH becomes particularly significant at lower dose ranges (≤ 0.5 Gy), where the accurate quantification of rare aberrations is crucial for reliable dose estimation yet most challenging for conventional methods^5,8^. In addition to improved sensitivity, pan-cent-FISH also demonstrates superior reproducibility by reducing observer bias, a critical advantage when comparing results across laboratories or over extended monitoring periods^5–9^.

The growing importance of precise biodosimetry in contemporary radiation protection emphasizes the need for rigorous comparison of emerging and established techniques^6^. Although some studies have demonstrated the theoretical and practical advantages of pan-cent-FISH^7–13^, the present investigation aims to systematically evaluate whether this technique enhances the precision and reliability of cytogenetic dose estimation within low-to-moderate dose ranges relevant to occupational radiation exposures and radiation-safety monitoring. Furthermore, the study compares its performance with conventional Giemsa-stained dicentric assays using controlled ex vivo irradiations to determine its potential applicability in operational biodosimetry. The practical validation is particularly notable when considering the full spectrum of biodosimetry applications, from routine occupational monitoring to emergency response^1,2^.

This study systematically compares pan-cent-FISH and conventional uniform Giemsa staining for radiation dose assessment using DC + R. We establish dose-response curves after controlled ex vivo ^60^Co-γ-irradiation, evaluate sensitivity across 0–3 Gy, and validate performance via blinded dose reconstruction. Beyond aberration detection efficiency, we assess practical utility for occupational monitoring and emergency triage. By quantifying improvements in dose-response linearity, precision, and accuracy, we provide actionable criteria for method selection. Our findings support laboratories adopting pan-cent-FISH and advance radiation protection practices amid growing demand for precise exposure assessment.

## Materials and methods

### Chemicals

L-glutamine and uniform Giemsa stain were procured from Sigma-Aldrich, USA. RPMI 1640, phytohemagglutinin (PHA), cytochalasin B (Cyto-B), fetal calf serum (FCS), streptomycin, penicillin, and colcemid were obtained from Gibco Life Technologies, USA. DPX was sourced from Merck, USA, and pan-centromeric probes were supplied by Metasystems, German.

### Ethical approval and blood collection

This study was approved by the Institutional Ethics Committee of BARC, Mumbai, India (Reference NO: BHMEC/NP/11/2024). Informed consent was obtained from all participants prior to peripheral blood collection. Four volunteers were recruited for the study: three contributed samples for generating the dose-response curve, and one volunteer participated in the blind dose-estimation experiment. A total of 21 mL of blood was collected in heparinized vacutainers from each of the three volunteers for dose–response analysis, and 4 mL blood was collected from the fourth volunteer for blind dose estimation. All experimental procedures were carried out in accordance with the guidelines and recommendations of the ethics committee.

### Radiation exposure, dose delivery, and calibration

After collection, peripheral blood samples from each individual were aliquoted into seven tubes and irradiated over a dose range of 0–3 Gy using ^60^Co-γ radiation at a dose rate of 0.4 Gy/min. All exposures were performed using a Blood Irradiator-2000 (BRIT, DAE, India). The irradiation procedure strictly adhered to established guidelines^1,2^. A water-equivalent PMMA phantom (3 mm build-up) was placed above the sample tubes to achieve electronic equilibrium and ensure accurate dose delivery. The phantom and blood samples were pre-warmed and maintained at 37 °C throughout irradiation to mimic physiological conditions and minimize temperature-related variability. Dose and dose-rate measurements were carried out using a Fricke dosimeter under identical irradiation geometry. The dosimetry was calibrated and traceable to national standards, with consistency cross-checked against international primary standards, ensuring high accuracy and reproducibility. All irradiations for a given calibration set were completed on the same day to avoid fluctuations in instrument performance and dose-rate stability.

### Blood culture, metaphase harvesting, uniform Giemsa staining, and dicentric scoring

Forty-two sets of whole blood cultures were established, six set of cultures (three sets for pan-cent-FISH and the remaining three sets for Giemsa staining) prepared for each of the seven dose points from the subject. The procedure followed the in-house optimized protocols, adhering to IAEA and ISO recommendations^1,2,14^. Each culture contained 4.5 mL of RPMI-1640 medium, 0.5 mL of fetal bovine serum (FBS), 0.1 mL of phytohemagglutinin-M (PHA-M), and 0.5 mL of heparinized peripheral blood. The cultures were incubated at 37 °C in a 5% CO_2_ environment for 52 h. To arrest cells in metaphase, colcemid (0.2 µg/mL) was added after 24 h. The cultures were then centrifuged at 186 g for 8 min, and the resulting pellet was treated with 0.075 M hypotonic KCl for 20 min. Following another round of centrifugation, the cells were fixed using Carnoy’s solution, with the fixation process repeated three times. Slides were prepared by dropping the cell suspension onto a glass slide from a height of 15 cm. Two slides were prepared for each dose point, one for uniform Giemsa staining and the other for pan-cent-FISH staining. The slide was stained with 10% uniform Giemsa solution and mounted using DPX. Approximately, 500 uniform Giemsa-stained metaphases were analyzed for dicentric chromosomes in accordance with IAEA and ISO guidelines^1,2^.

### Fluorescence in-situ hybridization (FISH) for Pan centromere staining (pan-cent-FISH)

For pan-cent-FISH analysis, fluorophore-labelled pan-centromeric probes from Metasystems (Germany) were applied to hybridise the centromeres of all chromosomes with slight modifications^15,16^. Briefly, after a brief pepsin treatment, 16 µL of the probe mixture was applied to each slide, denatured at 75 °C for 3 min, and hybridized for 4–5 h at 37 °C in a humidified chamber. The slide was then washed in 0.4× SSC at 72 °C (in a water bath), briefly rinsed in 2× SSC containing 0.05% Tween 20, and dehydrated in a graded ethanol series (80%, 90%, 100%). DAPI with antifade was subsequently applied for microscopic visualization.

### Image acquisition and aberration analysis

Slides were scanned and analyzed using an Axio Imager Z2 automated microscope (Carl Zeiss, Germany) equipped with a Cool Cube 5 camera and MetaSystems software (ISIS, Ikaros, Metafer5). For Giemsa-stained slides, image acquisition was performed using Metafer 5 in automated (auto-capture) mode; however, all aberration analysis was conducted manually by carefully reviewing the captured metaphase images. In any instance of ambiguity or uncertainty, the same metaphase was re-examined directly under the microscope to ensure correct identification. Automated dicentric scoring was not used in this study.

For pan-centromeric FISH, imaging was performed in automated mode using the ISIS module of the MetaSystems platform, while the analysis was carried out manually within the same module.

### Blind dose estimation

An anonymous, 29-year-old male volunteer was recruited for blind dose estimation. A 4 mL blood sample was collected and divided into four aliquots of 1 mL each, which were irradiated with four blinded doses, designated BD1 to BD4. Two sets of cultures were prepared for each blinded dose. Blood culturing, metaphase harvesting, and slide preparation were performed as described in “Blood culture, metaphase harvesting, uniform Giemsa staining, and dicentric scoring”, “Fluorescence in-situ hybridization (FISH) for pan centromere staining (pan-cent-FISH)”, and “Image acquisition and aberration analysis”. One set of slides was stained using pan-cent-FISH, while the other set was stained with uniform Giemsa stain for the scoring of “DC + R”. Scoring was conducted in accordance with the recommendations of the IAEA and ISO^1,2^. A total of ~ 1100 metaphases were analyzed for BD3, due to the low frequency of observed events, while ~ 700 metaphases were analyzed for each of the other samples (BD1, BD2, and BD4).

### Statistical analysis

Statistical analysis was carried out followed IAEA guidelines using Poisson statistics for aberration yield confidence intervals, dispersion index and Papworth u-test (σ^2^/Y ratios, u-tests)^1,2^. Dose-response curves were generated via weighted least squares regression (Dose Estimate v5.2), evaluated through χ^2^ and R^2^^17^. Method comparisons employed t-tests and ANOVA. Blind validation used relative error and mean absolute difference (MAD). All tests were two-tailed (α = 0.05).

## Results

As the aim of the study was to conduct a comparative evaluation of the detection and quantification of “DC + R” chromosomes in metaphases processed with pan-cent-FISH and uniform Giemsa staining, dose-response curves were first generated for both techniques, followed by blind dose estimations to assess their precision and reliability.

### Establishment of dose response curve for ^60^Co-γ radiation induced “DC + R” using pan-cent-FISH

**Fig. 1:**
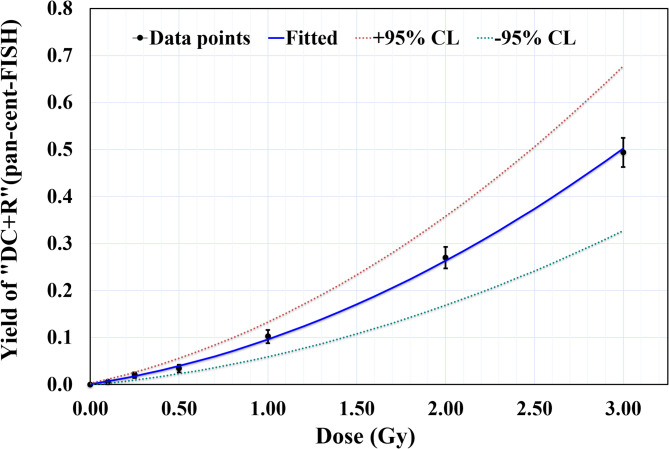
Dose-response curve for ^60^Co γ-ray-induced “DC + R” chromosomes as a function of radiation dose (0–3 Gy). “DC + R” chromosomes were visualised using pan-cent-FISH. The data were fitted to a linear-quadratic model (Y = C + αD + βD^2^). Error bars represent 95% confidence intervals (± 1.96σ).

**Fig. 2:**
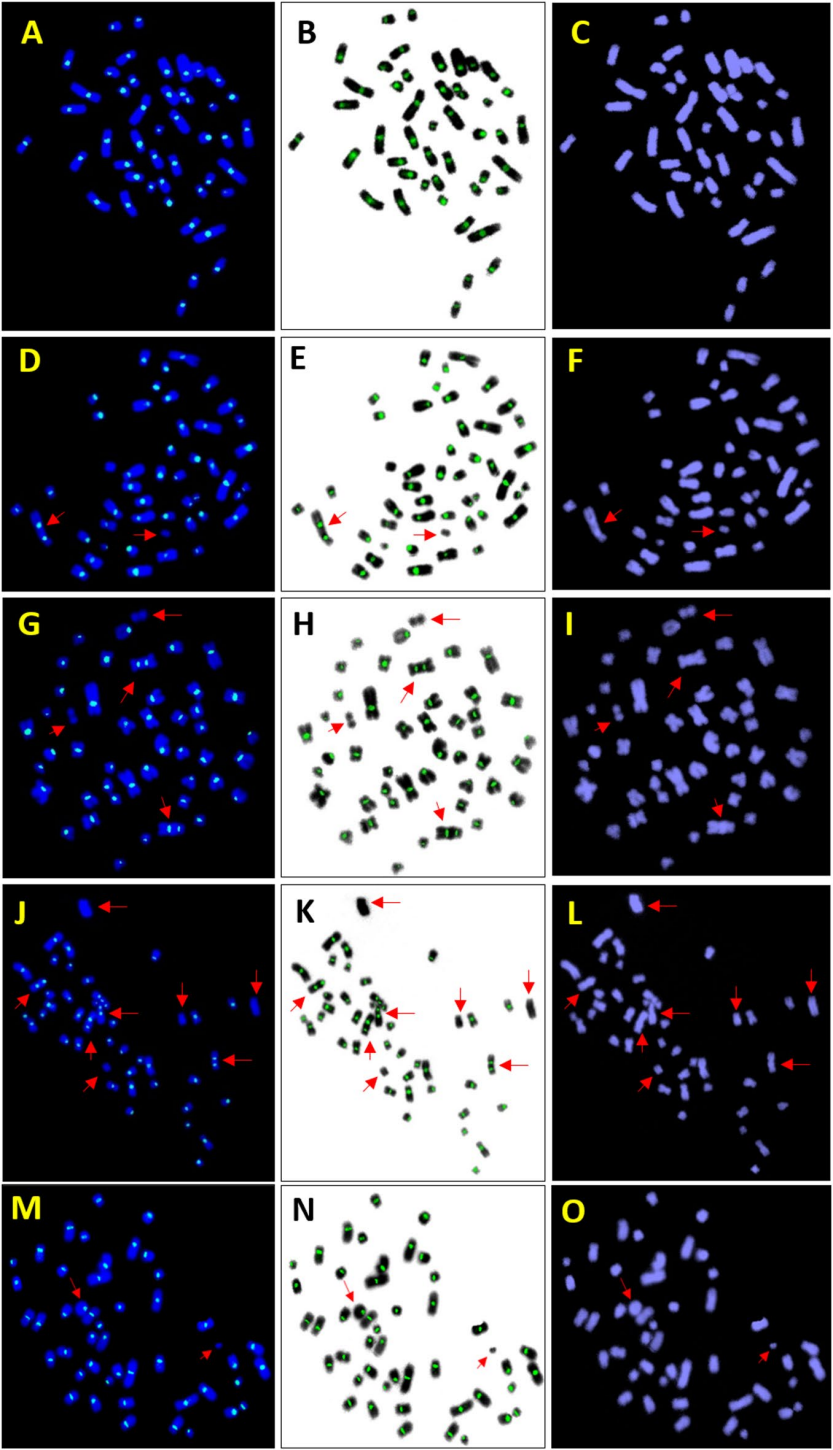
Pan-cent-FISH stained metaphase spreads illustrating cells with varying numbers of DCs. The left column displays merged images of blue (DAPI-chromosome) and green (Alexa flor 488 - centromere) fluorescence signals, the middle column shows merged green fluorescence (centromere) and inverse DAPI signals, and the right column presents DAPI-stained images alone. Panels (*A*–**C**) depict a normal cell with no DCs. Panels (**D**–**F**) depict a cell containing one DC. Panels (**G**–**I**) depict a cell with two DCs. Panels (**J**–**L**) depict a cell containing four DCs. Panels (**M**–**O**) depict a cell containing a ring (centric).

A dose-response curve for ^60^Co-γ radiation induced “DC + R” was generated using blood samples from three volunteer (Fig. 1). Centromeres were distinctly visualized with pan-cent-FISH staining (Fig. 2)^4^. The dose range was 0–3 Gy, delivered at a dose rate of 0.4 Gy/min. As shown in Table 1, a total of 15,043 metaphase spreads were analyzed, identifying 1217 “DC + R” chromosomes. Metaphase selection criteria adhered to IAEA and ISO standards, including spreads with DC and fragments or rings with fragments, while excluding those with only fragments or less than 46 centromeres^1,2^. The analysis showed a dose-dependent increase in cells with more than one “DC + R” chromosomes, with no such cells detected at doses ≤ 0.5 Gy. The first occurrence of a cell harbouring two “DC + R” chromosomes was observed at 1 Gy. Statistical evaluation confirmed a Poisson distribution of “DC + R” chromosomes at all dose points, with Papworth u test values falling within the range of ± 1.96. Table 1 provides a detailed summary of the number of metaphases analyzed, the distribution of “DC + R” chromosomes across dose points, and corresponding Papworth u test values and dispersion indices (σ^2^/Y).

The dose-response data were fitted to a linear-quadratic model (Y = C + αD + βD^2^), yielding a linear coefficient (α) of 0.0600 ± 0.0090 “DC + R” cell^− 1^, Gy^− 1^ and a quadratic coefficient (β) of 0.0364 ± 0.0050 “DC + R” cell^− 1^, Gy^− 2^. The model demonstrated a robust statistical fit, with a correlation coefficient (R^2^) of 0.999.

**Table 1:** Frequency and distribution of “DC + R” chromosomes in lymphocytes exposed to ^60^Co γ-rays in the dose range of 0–3 Gy. Centromeres were hybridized using fluorescent pan-centromere probes for accurate identification of “DC + R”.

Dose (Gy)	Cells scored	DC + *R*	Distribution of “Dicentrics” (Pan-centromeric-FISH)					Yield	Relative variance (σ2/Y)	Dispersion index (u)	SE
			D0	D1	D2	D3	D4				
0	3000	3	2997	3	0	0	0	0.001	0.999	− 0.032	0.021
0.1	3000	20	2980	20	0	0	0	0.0067	0.994	− 0.252	0.025
0.25	2005	42	1963	42	0	0	0	0.0209	0.98	− 0.655	0.031
0.5	2355	74	2281	74	0	0	0	0.0314	0.969	− 1.07	0.029
1	2478	260	2227	242	9	0	0	0.1049	0.965	− 1.24	0.028
2	1248	348	934	283	28	3	0	0.2788	0.935	− 1.64	0.04
3	957	470	573	308	67	8	1	0.4911	0.923	− 1.69	0.046

### Establishment of dose response curve for ^60^Co-γ radiation induced “DC + R” using uniform Giemsa staining

**Fig. 3:**
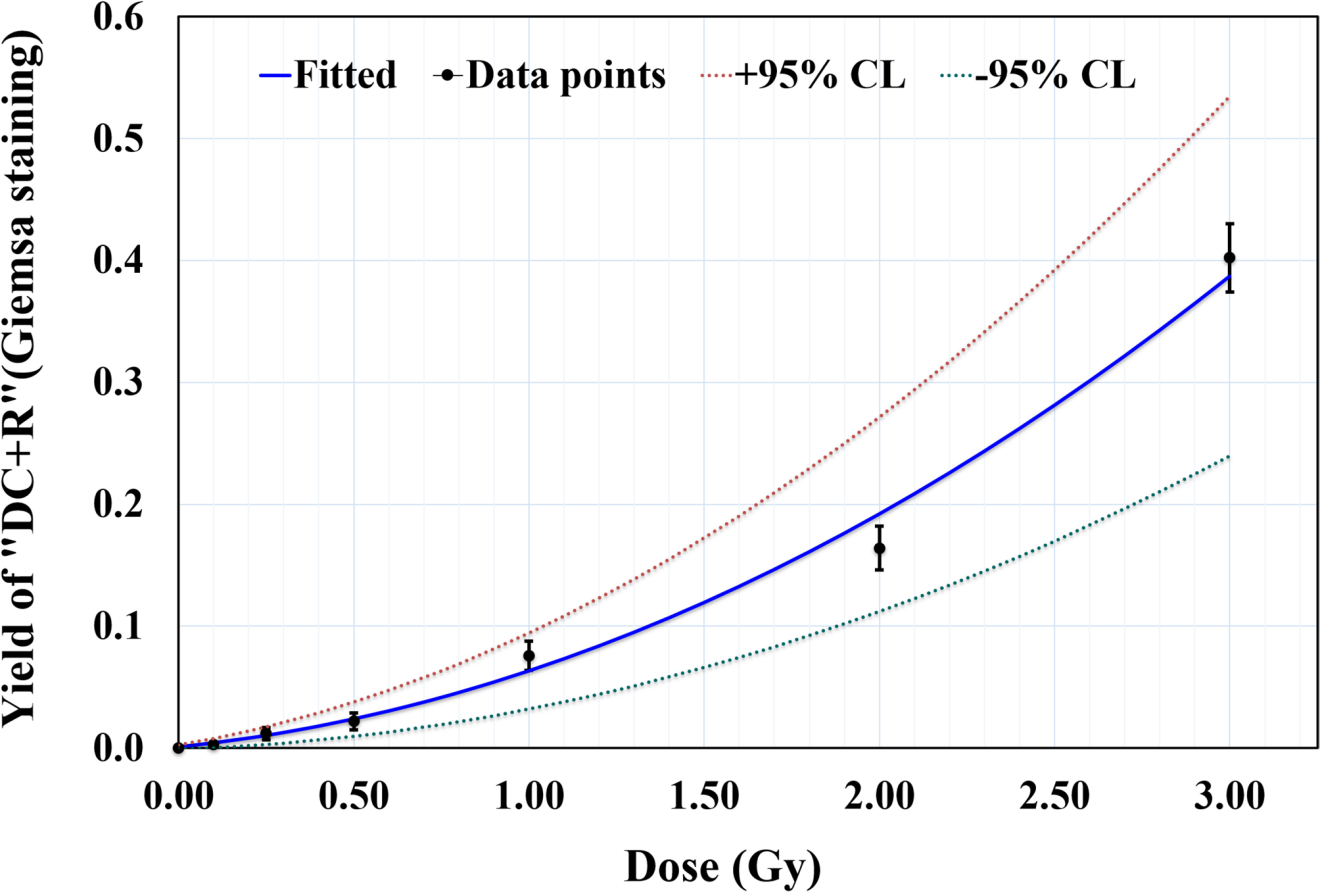
Dose-response curve for ^60^Co γ-ray induced “DC + R” chromosomes as a function of radiation dose (0 to 3 Gy), based on uniform Giemsa staining analysis. The curve is fitted with a linear-quadratic model (Y = C + αD + βD^2^), with a linear coefficient (α) of 0.0349 ± 0.0089 “DC + R” chromosomes cell^− 1^ Gy^− 1^ and a quadratic coefficient (β) of 0.0321 ± 0.0052 “DC + R” chromosomes cell^− 1^ Gy^− 2^. The error bars shown correspond to 95% confidence (1.96σ).

**Fig. 4:**
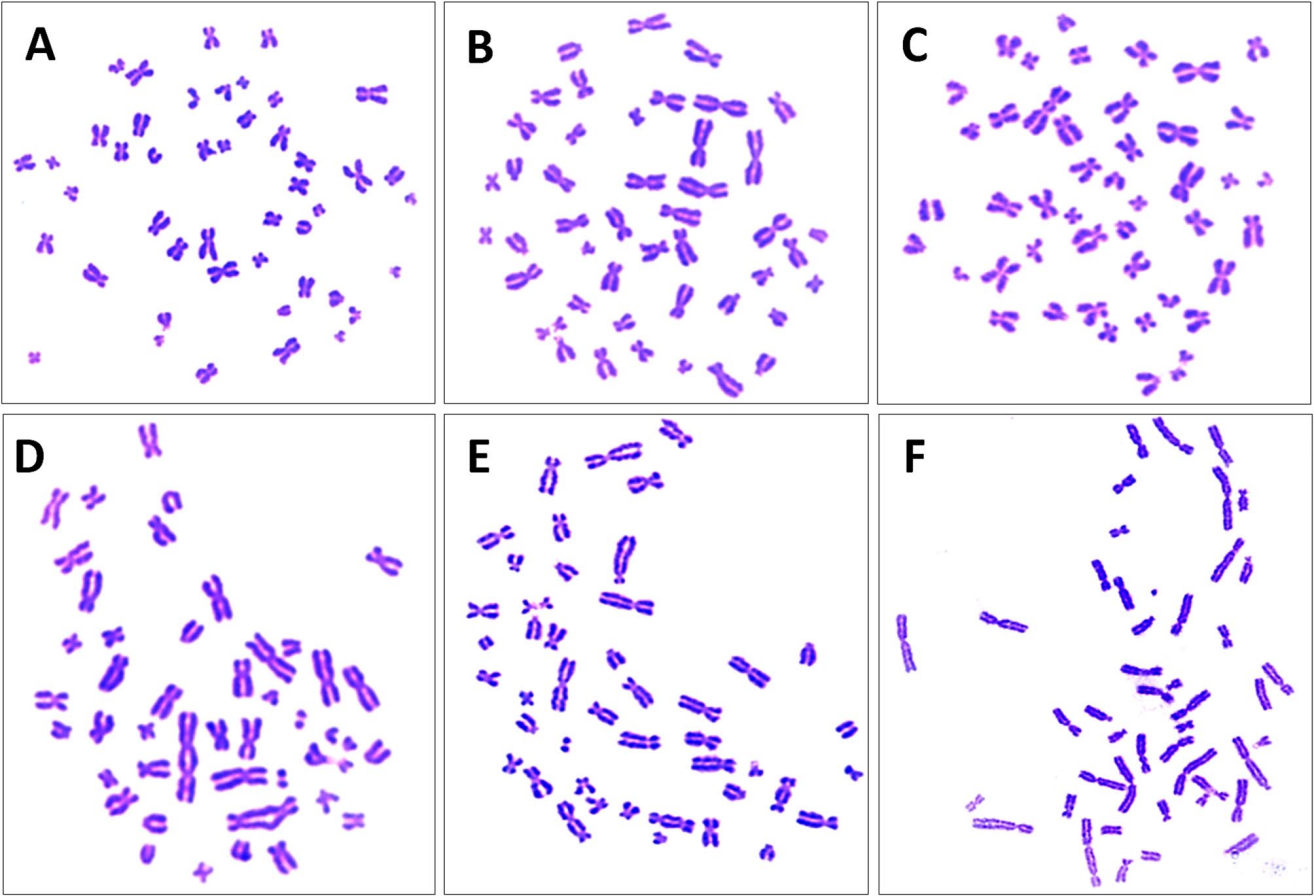
Uniform Giemsa-stained metaphases. Panels (**A**,**B**) display normal metaphases. Panel (**C**) shows a metaphase with one dicentric chromosome, (**D**) shows a metaphase with two dicentric chromosomes, (**E**) shows a metaphase with three dicentric chromosomes, and (**F**) shows a metaphase with four dicentric chromosomes.

The dose-response curve was established for uniform Giemsa-stained metaphase spreads using blood samples from the same three individual within the same dose range (0–3 Gy) (Figs. 3 and 4). The analysis involved 15,917 metaphase spreads, identifying 955 “DC + R” (Table 2). Metaphase selection criteria adhered to IAEA and ISO standards, as outlined previously^1,2^. Consistent with the findings from pan-cent-FISH, a dose-dependent increase in cells with more than one “DC + R” was observed, the first occurrence of a cell containing two “DC + R” were detected at 1 Gy. Statistical analysis confirmed that the distribution of “DC + R” chromosomes adhered to a Poisson distribution across all dose points. Table 2 summarizes the number of metaphases analyzed, the distribution of detected “DC + R” across dose points, and the corresponding Papworth u test values and dispersion indices (σ^2^/Y) in accordance with IAEA guidelines^1^. The dose-response data for “DC + R” were fitted to a linear-quadratic model (Y = C + αD + βD^2^), yielding a linear coefficient (α), 0.0349 ± 0.0089 “DC + R” cell^− 1^, Gy^− 1^ and a quadratic coefficient (β), 0.0321 ± 0.0052 “DC + R” cell^− 1^, Gy^−^². The model exhibited a strong statistical fit, with a correlation coefficient (R^2^) of 0.996.

**Table 2:** Frequency and distribution of “DC + R” chromosomes in lymphocytes from three donor after ex vivo exposure to ^60^Co γ-rays (0.1–3.0 Gy), analyzed by uniform Giemsa staining.

Dose (Gy)	Cells scored	DC + R	Distribution of “DC + *R*” (uniform Giemsa staining)				Yield	Relative variance (σ^2^/Y)	Dispersion index (u)	SE
			D0	D1	D2	D3				
0	3000	2	2998	2	0	0	0.0007	1	− 0.018	0.018
0.1	3000	8	2992	8	0	0	0.0027	0.998	− 0.097	0.024
0.25	2500	32	2468	32	0	0	0.0128	0.988	− 0.446	0.028
0.5	2487	58	2429	58	0	0	0.0233	0.977	− 0.815	0.028
1	2500	195	2309	187	4	0	0.0780	0.963	− 1.3	0.028
2	1409	241	1189	201	17	2	0.1710	1.02	0.546	0.038
3	1021	419	667	294	55	5	0.4104	0.906	− 1.7	0.044

### Comparison of dose-response curves and correlation analysis

**Fig. 5:**
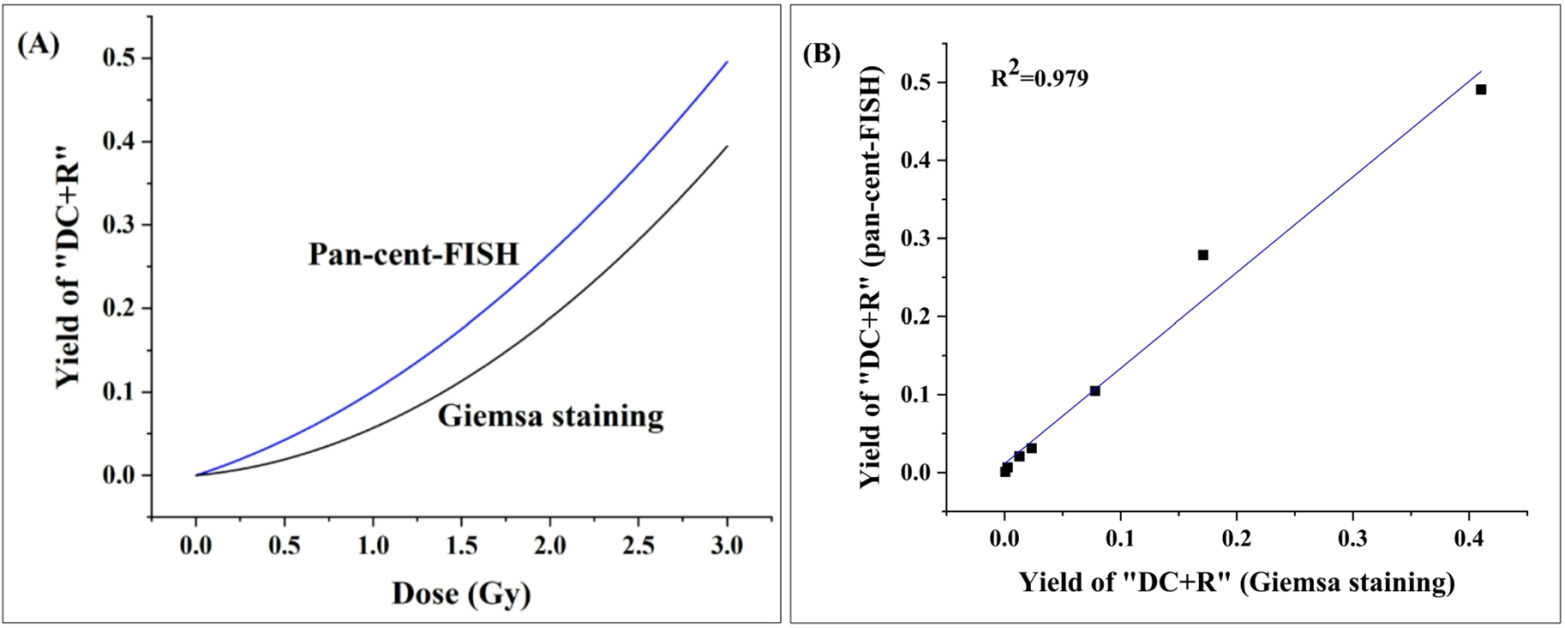
(**A**) Comparison of dose–response curves for “DC + R” identified using pan-cent-FISH and uniform Giemsa staining methods after ex vivo irradiation (0–3 Gy). (**B**) Correlation analysis of “DC + R” frequencies scored by pan-cent-FISH and uniform Giemsa staining across seven dose points (0, 0.1, 0.25, 0.5, 1, 2 and 3) in the dose–response curves. A slope of 1.23 ± 0.08 demonstrates that the yield of ‘DC + R’ measured by pan-cent-FISH is consistently higher than that measured by Giemsa staining, by a factor of 1.23.

Quantitative comparison of the dose-response parameters revealed significant differences between the two methodologies (Table 3; Fig. 5A). The linear coefficient (α) obtained through pan-cent-FISH analysis was approximately 1.7-fold greater than that derived from uniform Giemsa staining, demonstrating superior sensitivity in detecting linear dose-dependent increases in “DC + R” frequency. Similarly, the quadratic coefficient (β) showed a modest but consistent elevation in pan-cent-FISH analyses, indicating enhanced detection capability in the non-linear dose-response range. These findings, consistent with previous reports^5,9^, emphasize the technical advantages of pan-cent-FISH for accurate biodosimetry, particularly in the critical low-dose range (< 0.5 Gy) where precise aberration quantification is most challenging yet significant for regulatory purposes.

Correlation analysis between the two methodologies across seven dose points (0–3 Gy) demonstrated strong agreement (Fig. 5B). The coefficient of determination (R^2^ = 0.979) and Pearson’s correlation coefficient (*r* = 0.989, *p* < 0.001) both indicate an excellent linear relationship between “DC + R” yields measured by pan-cent-FISH and conventional uniform Giemsa staining. The standard error of estimate (0.071) confirms minimal deviation from the regression line, reflecting high measurement precision. While these results demonstrate good methodological concordance, the consistently higher aberration yields detected by pan-cent-FISH (particularly at low doses) highlight its superior sensitivity despite the strong correlation between techniques.

**Table 3:** Coefficients (α and β) and statistical parameters (chi-square, degrees of freedom, p-values for goodness of fit, and correlation coefficient) for “DC + R” detection using pan-cent-FISH and uniform Giemsa staining methods.

Method of scoring of “DC + *R*”	Linear Coefficient (α) ± SE (cell^− 1^ Gy^− 1^)	Quadratic Coefficient (β) ± SE (cell^− 1^ Gy^− 2^)	Background (C) (cell^− 1^)	Weighted chi-square value	Degrees of freedom	*p* value for goodness of fit	Correlation coefficient (“*r*” value)
Pan-cent-FISH	0.0600 (+/− 0.0090) (*p* = 0.0026)	0.0364 (+/− 0.0050) (*p* = 0.0019)	0.0009 (+/− 0.0008) (*p* = 0.2997)	8.4160	4	0.5387	0.9988
Uniform Giemsa staining	0.0349 (+/− 0.0089) (*p* = 0.0175)	0.0321 (+/− 0.0052) (*p* = 0.0034)	0.0005 (+/− 0.0007) (*p* = 0.5201)	13.59	4	0.5044	0.9958

### Validation of dose-response curve coefficients with blinded samples

**Fig. 6:**
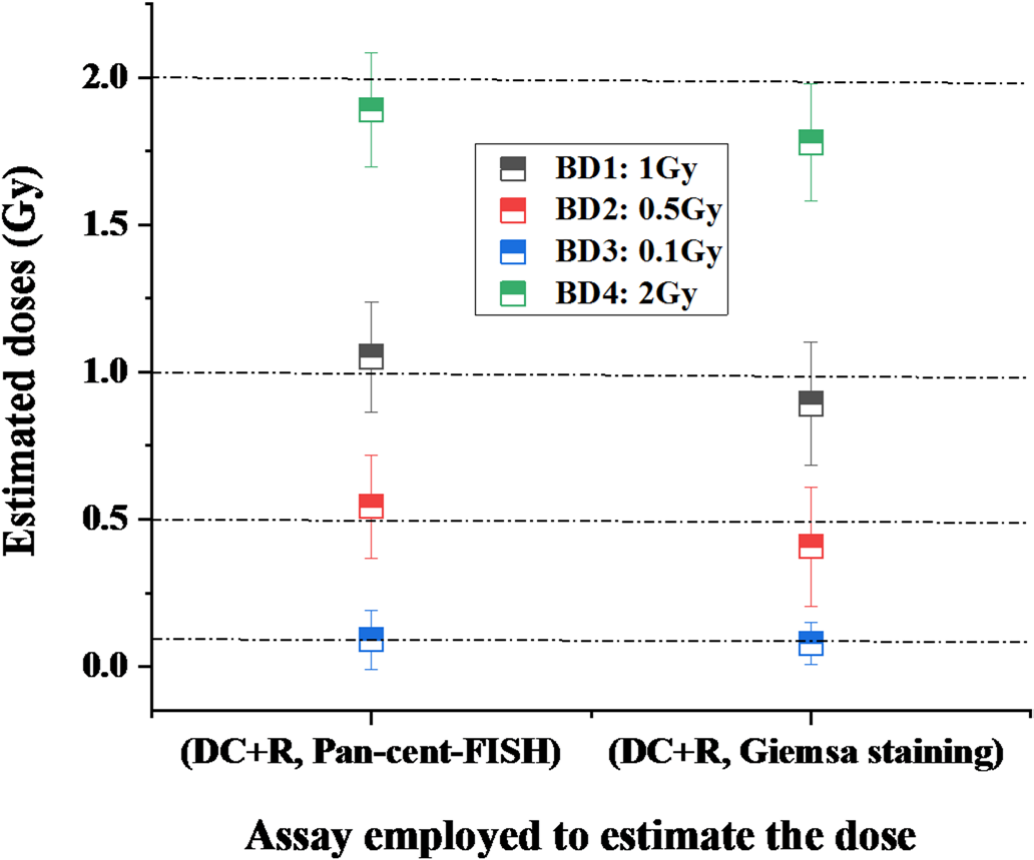
Graphical illustration of the estimated doses for eight blinded samples: 4 analyzed using pan-cent-FISH and 4 using uniform Giemsa staining for cytogenetic markers “DC + R”. Error bars represents 95% confidence intervals.

The established dose-response coefficients for both methodologies were validated through blinded dose reconstruction of eight test samples. As detailed in Table 4; Fig. 6, pan-cent-FISH demonstrated superior accuracy, particularly evident in the estimation of a 1 Gy dose (sample BD1(P)), which showed only 5.2% relative error - the most precise reconstruction in this study. In contrast, uniform Giemsa staining exhibited its largest deviation (21.0% relative error) when analyzing the 0.1 Gy sample (BD3(G)), highlighting its limitations in low-dose scenarios.

Comparative analysis revealed pan-cent-FISH consistently outperformed conventional staining across all test doses, with an average relative error of 7.13% versus 15.35% for uniform Giemsa staining (*p* < 0.01, paired t-test). This ~ 2-fold improvement in precision was further confirmed by mean absolute difference (MAD) analysis, where pan-cent-FISH achieved significantly lower deviation from true doses (0.0538 Gy vs. 0.1105 Gy; *p* < 0.001).

While both methods maintained 100% of estimates within the 95% confidence intervals - meeting minimum acceptability criteria for biodosimetry—the substantially enhanced precision of pan-cent-FISH, particularly at low doses (< 0.5 Gy) (in comparison to Giemsa staining method), strongly supports its adoption for applications requiring high-accuracy dose reconstruction.

**Table 4:** Validation of established dose-response curves by estimating the doses of 8 blinded samples: 4 analyzed using pan-cent-FISH and 4 using uniform Giemsa staining for cytogenetic markers “DC + R”.

Dose estimation using “DC + *R*” with pan-cent-FISH						
Blinded slide	True dose (Gy)	Aberration per cell	Estimated dose (Gy)	95% Lower confidence limit (Gy)	95% Upper confidence limit (Gy)	Relative error of the dose estimate (%)
BD1(P)	1	0.1043	1.052	0.879	1.239	+ 5.2%
BD2(P)	0.5	0.0443	0.544	0.393	0.719	+ 8.8%
BD3(P)	0.1	0.0067	0.091	0.029	0.192	-9.0%
BD4(P)	2	0.2443	1.89	1.705	2.083	-5.5%
Dose estimation using “DC + R” with uniform Giemsa staining						
BD1(G)	1	0.0571	0.892	0.702	1.101	-10.8%
BD2(G)	0.5	0.02	0.407	0.244	0.608	-18.6%
BD3(G)	0.1	0.0034	0.079	0.012	0.201	-21.0%
BD4(G)	2	0.1643	1.78	1.579	1.991	-11%

## Discussion

Conventional uniform Giemsa staining demonstrates substantial limitations in detecting “DC + R” aberrations, primarily due to its reliance on subjective morphological interpretation^1,2^. The manual scoring process introduces considerable inter-observer variability (CV = 15–20%) and reduced sensitivity, particularly at doses < 0.5 Gy where aberration frequencies are substantially low^1,18^. These constraints are particularly critical in occupational monitoring, where even minor dose estimation errors (± 0.1 Gy) can substantially influence radiation protection and regulatory decisions^1,2^. To overcome these limitations, we conducted a systematic comparison of pan-cent-FISH and conventional uniform Giemsa staining for “DC + R” quantification. Our results demonstrate that pan-cent-FISH exhibits superior sensitivity, detecting significantly more aberrations than uniform Giemsa across all dose levels (*p* < 0.001, Poisson regression). This difference was most striking in the low-dose range (< 0.5 Gy), where uniform Giemsa missed ~ 24–60% of the aberrations detected by pan-cent-FISH, with the highest deficit at 0.1 Gy. Although the performance gap narrowed at higher doses, pan-cent-FISH still detected ~ 11–31% more aberrations across 1–3 Gy (*p* < 0.05). Given this dose-dependent advantage, pan-cent-FISH emerges as a more reliable tool for biodosimetry, particularly in low-dose scenarios where detection sensitivity is critical. These findings align with M’Kacher et al. (2014 and 2015)^5,9^, who initially demonstrated the enhanced sensitivity of pan-cent-FISH for detecting DC in metaphases and premature condensed chromosomes (in PCC assay). More recently, Escalona et al. (2022) reported that pan-cent-FISH enhances the scoring efficiency and throughput of DC detection. Their study also showed that DC yields obtained using pan-cent-FISH were approximately 1.5–2-fold higher than those observed with conventional Giemsa staining in metaphase lymphocytes^12^. Our current metaphase-based analysis further validates this advantage, confirming its applicability to conventional chromosome spreads. Uniform Giemsa staining shows critical limitations at low doses (< 0.5 Gy), where its subjective morphology leads to significant errors - missing just 1–2 DCs/1000 cells causes > 20% dose estimation inaccuracies^1,2^. Dose-response curve further validated pan-cent-FISH’s superior performance: its 1.72 times higher linear coefficient (α) confirms enhanced low-dose sensitivity (*p* < 0.01), while the 1.13 times greater quadratic coefficient (β) improves high-dose detection.

In addition to methodological differences, the sensitivity and precision of cytogenetic biodosimetry assays are substantially influenced by the number of metaphases scored. Larger scoring pools greatly reduce statistical dispersion in aberration yields and allow more reliable discrimination between closely spaced dose points, especially in the low-dose range where aberration frequencies are inherently low. Therefore, increasing the number of scored metaphases enhances both dose estimation accuracy and confidence intervals, irrespective of the technique used. This principle is well established in biodosimetry guidelines and international recommendations, including the IAEA Cytogenetic Dosimetry Manual, which emphasizes that statistical power and assay sensitivity scale with the number of metaphases evaluated^1,2^.

In the present study, scoring was performed by a single observer, precluding evaluation of inter-observer variability. However, M’Kacher et al. (2014) previously demonstrated that pan-cent-FISH, combined with telomere staining, achieved markedly lower inter-scorer variability (coefficient of variation, CV = 8.6%) compared to conventional uniform Giemsa staining (CV = 18.7%), as reported by Romm et al. (2013), reflecting a 2.2-fold improvement in reproducibility^5,9,18^. This enhanced consistency highlights the suitability of centromere staining for high-precision biodosimetry, particularly in multi-institutional studies with diverse scorers. The molecular cytogenetic approach addresses well-documented limitations of uniform Giemsa staining, notably in detecting: (1) dicentrics involving acrocentric chromosomes (2), small interstitial dicentrics (< 2 Mb), and (3) dicentrics with centromere separation distances < 0.5 μm^5,9,12^. Pan-cent-FISH significantly improves detection efficiency for these challenging morphologies, which uniform Giemsa often misclassifies as monocentric aberrations due to its limited resolution (~ 5–10 Mb)^1,2^. The standardized fluorescence-based detection of pan-cent-FISH eliminates subjective morphological interpretation, reducing false negatives and establishing it as a robust and reliable method for precise radiation dose reconstruction.

The temporal requirements for dose estimation present distinct considerations across different exposure scenarios. In acute high-dose radiation events (> 1 Gy), where rapid triage is crucial for medical management (median required turnaround time < 72 h; IAEA, 2011), the extended processing time of cytogenetic biodosimetry (typically 72–96 h including lymphocyte culture) remains a significant constraint^1^. While conventional uniform Giemsa staining offers a shorter staining protocol (2–3 h vs. 4–5 h for pan-cent-FISH hybridization), our data demonstrate that pan-cent-FISH ultimately compensate the time through more efficient metaphase scoring (60 vs. 50 metaphases/hour; *p* < 0.05, t-test). The efficiency gains arise from its fluorescent centromeric labelling, which enables unambiguous aberration identification. This eliminates the need for repeated rescoring (reducing scorer dependence) and achieves 92% first-pass accuracy compared to 68% for uniform Giemsa-stained samples (*p* < 0.01)^19^. The standardized fluorescence signals minimize interpretive variability while maintaining analytical precision across users of varying experience levels. For occupational monitoring and diagnostic scenarios involving low-dose exposures (< 0.5 Gy), where precision in dose estimations is paramount, pan-cent-FISH’s enhanced sensitivity and reproducibility^5,9^.

The validation of dose-response relationships through blinded dose reconstruction demonstrated the superior performance of pan-cent-FISH across regulatory relevant dose range (< 0.5 Gy). Statistical analysis revealed significantly enhanced accuracy of pan-cent-FISH compared to conventional uniform Giemsa staining (*p* < 0.01, ANOVA), with a mean 2.2-fold reduction in relative error (7.13% versus 15.35%). This technical advantage was particularly evident at lower doses, where pan-cent-FISH achieved a relative error of 9.0% at 0.1 Gy compared to 21.0% for uniform Giemsa staining, exceeding the ICRP-recommended 15% error^20^ for reliable dose estimation in occupational monitoring scenarios. The precision of pan-cent-FISH was consistently maintained throughout the tested dose range, demonstrating relative errors of 8.8% at 0.5 Gy, 5.2% at 1.0 Gy, and 5.5% at 2.0 Gy, representing significant improvements over uniform Giemsa staining’s performance at each respective dose level (18.6%, 10.8%, and 11.0%). Further validation through MAD analysis confirmed these findings, with pan-cent-FISH yielding significantly lower values (0.0538 Gy vs. 0.1105 Gy; *p* < 0.001), corresponding to a ~ 51% improvement in dosimetric accuracy. The consistency of these results across multiple validation metrics, including relative error, MAD, and dose reconstruction accuracy, provides compelling evidence for the technical superiority of pan-cent-FISH.

While uniform Giemsa-based dicentric scoring was automated over a decade ago^21,22^, current efforts focus on developing commercial level high-throughput pan-cent-FISH platforms to overcome manual analysis limitations. The implementation of automated aberration scoring platforms holds significant promise for alleviating the considerable labor demands associated with manual analysis, a critical factor in large-scale radiation emergencies where rapid biodosimetry is essential for effective triage. The integration of high-throughput FISH systems has the potential to substantially enhance processing capacity, enabling timely and efficient assessment of mass casualties and facilitating optimized medical response strategies. A decade ago, M’Kacher et al. has developed (prototype) TCScore, an automated image analysis software which was capable of detecting dicentric chromosomes in telomere- and centromere-stained metaphases across diverse image formats^5^. Their validation studies demonstrated that TCScore achieved 95% concordance with manual scoring for dicentric identification, significantly improving throughput while maintaining analytical accuracy (*p* < 0.001, Pearson correlation).

A comparative cost-benefit analysis is essential when evaluating methodologies serving the same objective. Regarding DC detection, uniform Giemsa staining presents a more economical approach concerning both reagents and standard cytogenetics instrumentation. Conversely, pan-cent-FISH requires a significantly greater financial investment, primarily due to the expense of specialized fluorescent probes and the necessity of advanced equipment, including a fluorescence microscope and, optimally, an image analysis system. While FISH’s higher costs may be justifiable for improved accuracy in dose estimations, uniform Giemsa remains the practical choice for resource-limited settings.

Despite the strengths of the present work, several areas offer opportunities for further refinement. While three donors were included in this study, expanding the cohort size in future investigations would enhance the statistical robustness of the calibration curves and improve the generalizability of the findings across inter-individual variability. The number of metaphases scored per dose point, although compliant with IAEA/ISO recommendations, can be further increased to reduce statistical uncertainty, particularly in the low-dose region^1,2^. All experiments were conducted within a single laboratory, using the same irradiation setup, culture conditions, and imaging systems. Multi-laboratory validation is needed to assess the reproducibility and transferability of the calibration curves under varying operational conditions. Scoring was performed by a limited number of trained analysts, which may introduce subtle scorer-dependent bias. Increasing the number of independent scorers and conducting inter-scorer variability assessments would strengthen confidence in aberration identification, especially for borderline or complex dicentrics. Although pan-centromeric FISH offers clear advantages, future work should evaluate performance under more diverse exposure conditions to better reflect real-world scenarios. In addition, the calibration curves were established under ex vivo acute, homogeneous irradiation conditions, which may not fully represent in vivo exposure scenarios such as partial-body, protracted, or low dose-rate exposures, potentially affecting dicentric yields and dose-response relationships when applied to real-world situations.

In the present study, the calibration curve was established up to 3 Gy, and future work aims to extend the dose range to 5 Gy to strengthen the high-dose segment and broaden the applicability of the assay for diverse radiation exposure scenarios. The Biodosimetry Laboratory at Bhabha Atomic Research Centre (BARC), India, serves as the national reference facility for radiation biodose assessment^23–25^, and the dose-response curves generated in this study will contribute to standardized calibration references for the country. Ongoing efforts are directed toward expanding the dataset with additional volunteer samples to further enhance the robustness and reliability of this reference curve.

## Conclusion

The findings of this study demonstrate the advantage of pan-cent-FISH over uniform Giemsa staining for biodosimetric applications, particularly in detecting and quantifying radiation-induced “DC + R”. The dose-response curves generated using pan-cent-FISH exhibited higher sensitivity, with around two-fold increase in the linear coefficient and improved detection in the quadratic range compared to uniform Giemsa staining. Statistical analyses confirmed a strong correlation between the two methods, although pan-cent-FISH achieved better precision, as evidenced by a lower MAD compared to uniform Giemsa staining. Furthermore, blinded dose estimation validated the higher accuracy and lower average relative error of pan-cent-FISH relative to uniform Giemsa staining. These results highlight the better applicability of pan-cent-FISH for precise dose assessment, making it more suitable for regulatory compliance and radiological emergency response.

## Data Availability

All data generated in this study are included in the article. Additional data supporting the findings are available from the corresponding author (Email ID: nageshnb@barc.gov.in), upon reasonable request.
